# Calcium Channel Blockers, Progression to Dementia, and Effects on Amyloid Beta Peptide Production

**DOI:** 10.1155/2015/787805

**Published:** 2015-06-28

**Authors:** Mark A. Lovell, Erin Abner, Richard Kryscio, Liou Xu, Shuling X. Fister, Bert C. Lynn

**Affiliations:** ^1^Department of Chemistry, University of Kentucky, Lexington, KY 40506, USA; ^2^Sanders-Brown Center on Aging and Alzheimer's Disease Center, University of Kentucky, Lexington, KY 40536, USA; ^3^Department of Epidemiology, University of Kentucky, Lexington, KY 40506, USA; ^4^Departments of Statistics and Biostatistics, University of Kentucky, Lexington, KY 20536, USA; ^5^National Minority Quality Forum, Washington, DC 20005, USA

## Abstract

Previous epidemiologic studies suggest that antihypertensive drugs may be protective against cognitive decline. To determine if subjects enrolled in the University of Kentucky longitudinal aging study who used antihypertensive drugs showed diminished progression to dementia, we used a 3-parameter logistic regression model to compare the rate of progression to dementia for subjects who used any of the five common categories of antihypertensive drugs to those with similar demographic characteristics but who did not use antihypertensives. Regression modeling showed that subjects who used calcium channel blockers (CCBs) but not the other classes of antihypertensives showed a significant decrease in the rate of progression to dementia. Significantly, use of CCBs ameliorated the negative effects of the presence of APOE-4 alleles on cognitive decline. To determine if CCBs could minimize amyloid beta peptide (A*β*_1–42_) production, H4 neuroglioma cultures transfected to overexpress APP were treated with various CCBs and A*β*_1–42_ levels and levels of proteins involved in A*β* production were quantified. Results show that treatment with nifedipine led to a significant decrease in levels of A*β*_1–42_, with no significant decrease in cell viability. Collectively, these data suggest that use of CCBs significantly diminishes the rate of progression to dementia and may minimize formation of A*β*_1–42_.

## 1. Introduction

Alzheimer's disease (AD) is the 6th leading cause of death in the United States and today affects 5.2 million Americans aged 65 and more [1]. Without preventive strategies or development of an efficacious treatment, there may be 16 million Americans with AD by the year 2050 [1].

Pathologically, AD is characterized by an abundance of neurofibrillary tangles (NFT), senile plaques (SP), neuropil thread formation, and A*β* deposition; neuron and synapse loss; and proliferation of reactive astrocytes, particularly in the hippocampus, amygdala, entorhinal cortex, and neocortex. NFT are composed of intracellular deposits of paired helical filaments composed of hyperphosphorylated tau. Senile plaques are present as diffuse plaques composed of amorphous extracellular deposits of amyloid beta (A*β*) that lack neurites and neuritic plaques (NP) composed of extracellular deposits of insoluble A*β* surrounded by dystrophic neurites, reactive astrocytes, and activated microglia. In addition to insoluble A*β* present in SP, soluble A*β* oligomers are present in the AD brain and may represent the main toxic form of A*β*, thus implicating them in the disease process [2–4].

Currently, two classes of medications are FDA approved for use by AD patients including cholinesterase inhibitors (Aricept) and an N-methyl-D-aspartate (NMDA) antagonist (Memantine). Although these therapeutics show clinical benefit in some patients, many do not respond. Additionally, these drugs do not significantly modify disease progression and perhaps more importantly are not approved for patients at earlier stages of the disease (mild cognitive impairment; MCI). For these reasons there is a critical need to identify additional therapeutics that can be initiated early in disease progression to alter the pathogenesis of the disease. To date, most experimental therapeutics have focused on modulation of the major pathologic features of the AD brain by designing drugs that decrease formation of the 42-amino acid amyloid beta peptide (A*β*_1–42_) or modify its capacity for formation of neurotoxic oligomers or the modulation of tau hyperphosphorylation and NFT formation through manipulation of kinases responsible for tau phosphorylation.

Although several therapeutic compounds have been developed and tested for use in AD, none to date have proven useful in the modulation of the pathogenesis of AD and the cognitive decline associated with disease progression. In an attempt to identify drugs commonly used by elderly patients that may diminish cognitive decline, multiple retrospective epidemiologic studies have been carried out. One class of potentially beneficial drugs identified is antihypertensives (AHTs). Recent prospective cohort studies suggest that hypertension in midlife is associated with increased risk of dementia in later life [5–12] and increased risk of hippocampal atrophy [13]. Multiple studies suggest that various AHTs may be effective in slowing cognitive decline although it remains unclear if protective effects observed are due to cardiovascular protection. To determine if volunteers enrolled in the University of Kentucky Alzheimer's Disease Center (UK-ADC) Biologically Resilient Adults in Neurological Studies (BRAiNS) cohort prescribed AHTs showed slower progression to dementia compared to subjects not taking AHTs, we used a three-parameter logistic model to analyze cognitive and clinical data maintained by the UK-ADC Biostatistics Core. In addition, we tested the effect of various AHTs on formation of amyloid beta peptide (A*β*_1–42_) in cell culture.

## 2. Materials and Methods

### 2.1. Case-Control Study

**Figure 1:**
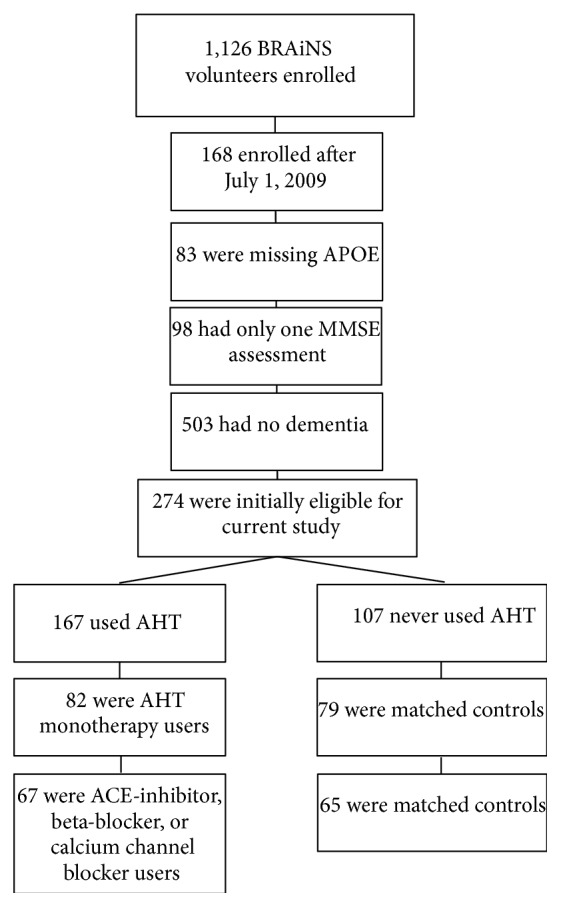
Flow chart demonstrating subject stratification leading to subjects included for analysis.

Subjects of this study were drawn from volunteers in the BRAiNS cohort at the UK-ADC, a longitudinal cohort of approximately 1,100 individuals established in 1989 with ongoing recruitment [14] as shown in Figure 1. The cohort comprises a convenience sample of older adults (age ≥ 60 years) from central Kentucky. Exclusion criteria for the BRAiNS cohort include prevalent neurological, psychiatric, and disabling medical disorders, as well as prevalent dementing illness (see [14] for a detailed listing and explanation of recruitment and procedures). Participants undergo annual cognitive and clinical assessments and donate their brains upon death. All volunteers were cognitively normal at study entry, and all research activities were approved by the University of Kentucky Institutional Review Board. Each participant provided written informed consent.

Initial inclusion criteria for the current study included at least two clinical assessments with an assessment of at least mild dementia during follow-up (MMSE ≤ 26) or clinical diagnosis of dementia and known APOE genotype (*n* = 274). Eligible subjects were identified who reported use of AHT medications from each of the 5 commonly prescribed categories (angiotensin converting enzyme (ACE) inhibitors, angiotensin II receptor blockers, beta-blockers, calcium channel blockers (CCBs), or diuretics; Table 1) and were designated as AHT-positive subjects (*N* for each category shown in Table 1). Participants who reported use of multiple AHT drugs were excluded. These AHT-positive subjects were matched as closely as possible to subjects who never reported taking AHTs (AHT-negative) by age of entrance into the study group, by gender, and as closely as possible by the number of MMSE assessments (taken before and after the age medications began). Subject demographic data are shown in Table 2. Cognitive and clinical data maintained by the UK-ADC Biostatistics Core were analyzed to determine if subjects using the 5 classes of commonly prescribed AHTs showed diminished cognitive decline measured by MMSE scores compared to subjects without AHT therapy. Because medical history data were not routinely collected until 2001, presence of hypertension could not be included in the analysis. Studies described in the paper were carried out in accordance with The Code of Ethics of the World Medical Association (Declaration of Helsinki) for experiments involving humans and were approved by the University of Kentucky Institutional Review Board.

### 2.2. H4 Survival and A*β* Measurement

To determine if CCBs lead to altered A*β* processing* in vitro*, H4 human neuroglioma cell lines transfected to overexpress the amyloid precursor protein (APP) (H4-APP cells) [15, 16] were maintained in Opti-MEM (Invitrogen) with 10% fetal bovine serum (FBS) and hygromycin B (0.2 mg/mL) and were split 1 : 2 every two days. For treatment, cells were plated at 2.5 × 10^5^ cells/well in 6-well plates and allowed to grow for 24 hours. Cultures were switched to Opti-MEM and treated with CCBs or vehicle (DMSO) for 16 hours. Following treatment, media were collected from each well, mixed with 5 *μ*L 50 mM EDTA, and flash-frozen until used for A*β* quantification. An equivalent volume of Opti-MEM was added to each well and cell viability assessed by adding 3-[4,5-dimethylthiazol-2yl]-2,5-diphenyl tetrazolium bromide (MTT) (500 *μ*g/mL final concentration) as a measure of cell viability.

### 2.3. A*β* ELISA

Levels of A*β*_1–42_ secreted into the medium were quantified using standard sandwich ELISAs (Invitrogen) as per manufacturer's instructions. Data are reported as the mean ± SEM % control (vehicle) A*β*_1–42_.

### 2.4. Western Blot Analysis

For quantification of proteins involved in A*β* processing H4 cells were plated at 2.5 × 10^5^ cells/well in 6-well plates and allowed to grow for 24 hours. Based on results of survival studies, cells were treated with 1 *μ*M nifedipine or vehicle for 16 hours. Following treatment cells were rinsed 3 times in PBS and cells from a single plate collected to make a single sample. Experiments were carried out using 6 to 9 samples (plates) per treatment. Cell pellets were homogenized in HEPES containing 137 mM NaCl, 4.6 mM KCl, 0.6 mM MgSO_4_, 0.7 *μ*g/mL pepstatin A, 0.5 *μ*g/mL leupeptin, 0.5 *μ*g/mL aprotinin, and 40 *μ*g/mL phenyl methyl sulphonyl fluoride, using an insulin syringe with a 26-gauge needle. Samples were centrifuged at 16,000 ×g for 10 minutes and the supernatant was used for western blot analyses. Protein samples (20 *μ*g) were boiled in 4x SDS loading buffer for 5 min and separated by electrophoresis on 4–15% gradient sodium dodecyl sulfate polyacrylamide gels, transferred to nitrocellulose membranes, and probed using antibodies against BACE-1 (R&D Systems; 1 : 1500 dilution; MW identified = 56 kDa), PS-1 (Cell Signaling; 1 : 750 dilution; MW = 55 kDa (full length)), NCT (Cell Signaling; 1 : 1500 dilution; MW = 120/110 kDa), cleaved Notch-1 (Novus; 1 : 1500; MW = 80 kDa), and ADAM-10 (presumed *α*-secretase; Santa Cruz; 1 : 1500 dilution; MW (active) = 60 kDa). Band intensities were quantified using a Li-Cor system and results normalized to levels of GAPDH on each gel. Results are expressed as mean ± SEM % vehicle.

### 2.5. Statistical Analysis

Since MMSE scores are known to decline with age and are bounded between 0 (floor) and 30 (ceiling), the scores were regressed on the age at which they were obtained using a three-parameter logistic regression model described below. The model has a random effect to account for the correlation among responses obtained from the same subject over time. All computations were done using the NLMIXED procedure in PC-SAS version 9.2.

The three-parameter logistic model used in the regression analysis was defined as

(1) $$\begin{array}{c} Y_{it}=\frac{a}{1+\exp\left\{b\left(t-c\right)\right\}}, \end{array}$$

where *Y*_*it*_ represents the MMSE score for the *i*th subject at age *t*. The parameter *a* is the asymptote for that subject, which varies by subject, the parameter *b* is a scaling effect representing 75% of the asymptote, and the parameter *c* is the midpoint of the curve or 50% of the asymptote. We assume that only the parameter *c* depends on the fixed effects while the parameter *a* depends only on the random effect and *b* is a scaling parameter. The fixed effects, or covariates of interest, are educational level, initial age, APOE-4 status, and AHT-positive/AHT-negative status. Since education did not influence the midpoint parameter (*P* > 0.10), it was dropped from the final model. The random effects are assumed to follow independent normal distribution (across subjects) with mean 30 (the ceiling) and unknown variance and are assumed to be the asymptote *a* in the model. The purpose of the modeling was to determine how each parameter depended on these covariates after accounting for the two sources of variability defined by the random effect: between- and within-subject variability.

Statistical analyses for cell culture studies were carried out using analysis of variance (ANOVA) with Dunnett's* post hoc* test for individual differences and the commercially available ABSTAT (AndersonBell, Arvada, CO, USA) software.

## 3. Results

Subjects were selected from the UK-ADC database who reported taking any of the 5 classes of AHTs following the flow diagram shown in Figure 1 and shown in Table 1. The database of MMSE scores consisted of 603 observations taken on 63 subjects (31 AHT-negative and 32 AHT-positive subjects) (one AHT-negative and two AHT-positive subjects had missing data on APOE-4 status and were omitted from the analysis) for subjects taking CCBs; 26 subjects (13 AHT-negative and 13 AHT-positive subjects; 200 observations) for subjects taking ACE inhibitors; and 43 subjects (21 AHT-negative and 22 AHT-positive subjects; 238 observations) for subjects on beta-blockers. Identification of subjects who took only diuretics (*N* = 9) or angiotensin II receptor blockers (*N* = 6) showed too few subjects to allow stratification by APOE-4 status, and they were not included in the analysis. After carrying out regression analysis for groups on each of the three drugs the resulting best fit of the data showed that the parameter *b* did not depend on any of the fixed effects while the parameter *c* depended on three covariates: initial age (*P* < 0.0001), APOE-4 status (*P* = 0.008), and AHT status (*P* = 0.01) for subjects taking CCBs. Since the parameter *c* is a linear combination of these three covariates, details on the beta estimate and standard errors can be found in Table 3. Subjects taking the other AHTs did not show significant effects on progression to severe global cognitive impairment.

**Figure 2:**
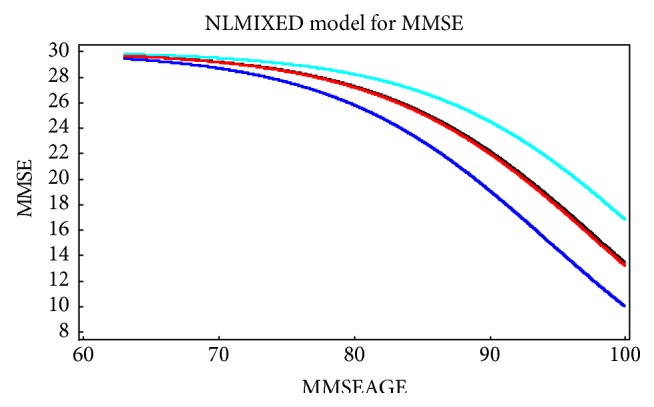
Plot of the estimated rate of decline in MMSE scores across the age span: top curve (light blue) shows patients who were APOE negative who used CCBs; the 2nd curve (black) shows APOE-4 negative patients without CCB use; the third curve (red) which lies just below the 2nd is for APOE-4 positive subjects who used CCBs; and the bottom curve (dark blue) is APOE-4 positive subjects who did not use CCBs.

To illustrate the fit of the model to the data, Figure 2 shows the trajectory of the fitted model versus age for subjects enrolled in the cohort at initial age 75. Based on Table 3 this trajectory varies by AHT status and APOE-4 status. The effect of each variable is about the same (a shift of 3.6 years for AHT-positive subjects compared to AHT-negative subjects and a shift of 3.8 years for no APOE-4 versus APOE-4 subjects). Specifically, the parameter *c* accounts for a shift at the midscore (MMSE score of 15) with a shift of roughly 3.7 years for each of these variables at that score. In other words, it takes an AHT-positive subject (those on CCBs) 3.6 years longer to attain the MMSE score 15 than those not on medications. As expected, the analyses showed that the presence of APOE-4 alleles led to an accelerated trajectory of progression to severe global cognitive impairment. In contrast, APOE-4 positive subjects who were prescribed CCBs showed a significant protective effect and a trajectory to dementia equivalent to that of subjects without APOE-4 alleles or CCB use. Additionally, subjects without APOE-4 alleles who reported use of CCBs showed significantly slower progression to severe global cognitive impairment (~4 years).

### 3.1. Cell Culture Studies

**Figure 3:**
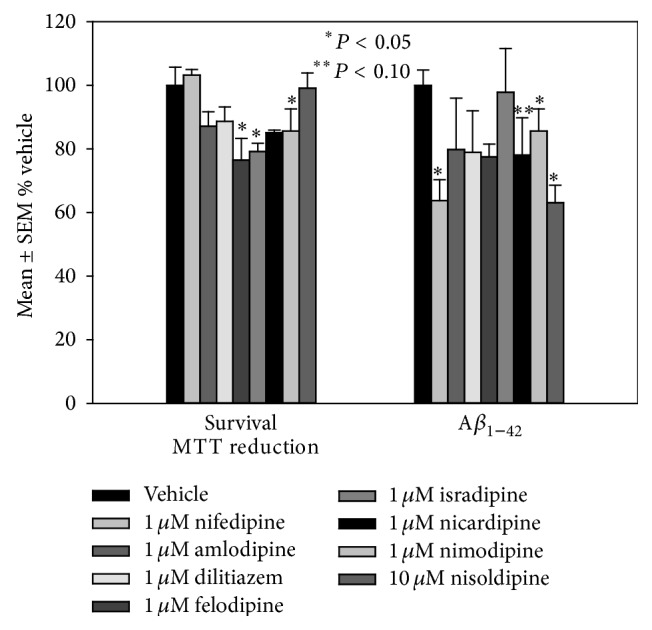
Mean ± SEM H4 survival and A*β*_1–42_ production after treatment with commonly prescribed CCBs. ^*∗*^ *P* < 0.05.

Because the regression model suggested that use of CCBs is associated with slowed progression to dementia we wanted to determine if CCB treatment significantly impacts pathogenic processes associated with AD. To determine if CCBs diminish A*β*_1–42_ formation we treated H4 neuroglioma cultures overexpressing APP with increasing concentrations of the 8 most commonly prescribed CCBs (amlodipine, dilitiazem, felodipine, isradipine, nicardipine, nimodipine, nifedipine, or nisoldipine) and measured A*β*_1–42_ secretion into culture medium. Cultures were treated with each drug for 16 hours and cell viability was assessed using the reduction of MTT.  Figure 3 shows that 1 *μ*M amlodipine, dilitiazem, felodipine, isradipine, nicardipine, and nifedipine led to minimal cell death whereas higher concentrations of nisoldipine (10 *μ*M) were well tolerated by the cultures. Using these optimized concentrations for further studies, we plated H4 cultures in 6-well plates at a density of 2.5 × 10^5^ cells/well and after 24 hours of equilibration exposed the cells to each agent (*N* = 9 to 18 over three experiments) for 16 h. Following treatment, medium was collected and mixed with 5 *μ*L 1 mM EDTA to inhibit metalloprotease activity and A*β*_1–42_ levels secreted into culture medium quantified using standard sandwich ELISAs (Invitrogen) as per manufacturer's instructions. Figure 2 shows that of the 8 CCBs tested 1 *μ*M nifedipine, 1 *μ*M nimodipine, and 10 *μ*M nisoldipine led to significantly lower A*β*_1–42_ generation with nifedipine showing the most pronounced change. It is interesting to note that nifedipine and nisoldipine are unique among CCBs in that both contain an ortho-nitro group whereas the other drugs contain a meta-nitro group.

**Figure 4:**
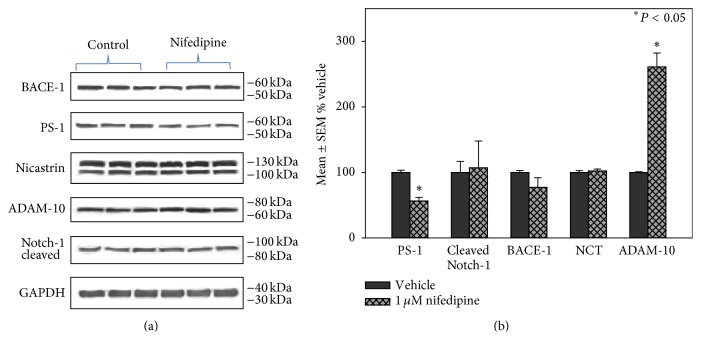
(a) Representative western blots for proteins involved in A*β* processing in H4 neuroglioma cells treated with nifedipine. (b) Levels of proteins involved in A*β* processing following treatment with nifedipine. Results are reported as mean ± SEM percent vehicle treated cells. ^*∗*^ *P* < 0.05.

Because nifedipine treatment led to the most pronounced decrease in A*β*_1–42_ production with minimal cell toxicity, we chose to use it for further study of the potential effects of representative CCBs on proteins involved in A*β* processing. For these studies, H4 cells were plated at a density of 2.5 × 10^5^ cells/well in 6-well plates and were exposed to 1 *μ*M nifedipine for 16 hours. Following exposure, the cells were rinsed in PBS and cells from 6 wells were combined to generate a single sample for protein analyses (*N* = 3 to 6 samples (plates) per treatment). Cells were homogenized using a micro-Dounce homogenizer and protein content was measured using the Pierce BCA method as per manufacturer's instructions. Protein samples (20 *μ*g) were subjected to SDS/PAGE using 5 to 20% linear gradient gels and transferred to nitrocellulose and probed with antibodies against BACE-1 (R&D Systems; 1 : 1550 dilution; MW identified = 56 kDa), PS-1 (Cell Signaling; 1 : 750 dilution; MW = 55 kDa (full length)), NCT (Cell Signaling; 1 : 1500 dilution; MW = 120/110 kDa), cleaved Notch-1 (Novus; 1 : 1500 dilution; MW = 80 kDa), and ADAM-10 (presumed *α*-secretase; Santa Cruz; 1 : 1500 dilution; MW (active) = 60 kDa).  Figure 4(a) shows representative western blots for each protein.  Figure 4(b) shows that treatment with nifedipine led to a statistically significant decrease of PS-1, and a significant increase in ADAM-10 compared to cultures treated with vehicle alone (DMSO) suggesting that the decrease in A*β* observed may be due to diminished *β*- and *γ*-secretase activity and increased *α*-secretase activity.

## 4. Discussion

Although several therapeutic compounds have been developed and tested for use in AD, none to date have proven useful in the modulation of the pathogenesis of AD and the cognitive decline associated with disease progression. In an attempt to identify drugs commonly used by elderly patients that may diminish cognitive decline, multiple retrospective epidemiologic studies have been carried out. One class of drugs identified is antihypertensives (AHTs). Several prospective cohort studies of 3290 subjects in Cache County Utah showed a lower incidence of AD in subjects taking AHTs at baseline (particularly potassium sparring diuretics) compared to those who did not [17]. More recently, a longitudinal study of 1810 patients aged 75 years and more in the Kungsholmen district of Stockholm, Sweden, showed that patients taking diuretics had a lower rate of dementia, whereas other AHTs including *β*-blockers and calcium channel blockers (CCBs) did not [18]. More recent randomized placebo controlled studies show that subjects over 60 years of age from 19 countries in Europe with no history of dementia who received active treatment with the CCB nitrendipine followed by enalapril or hydrochlorothiazide (*n* = 1238) had an 80% decreased risk of dementia or AD compared to placebo control (*n* = 1180) [19–22]. The Perindopril Protection Against Recurrent Stroke Study (PROGRESS), which included 6105 patients with a history of stroke or ischemia from 10 countries receiving perindopril and indapamide, showed a significant reduction in cognitive decline compared to those receiving placebo controls [23]. In contrast, the Study on Cognition and Progression in the Elderly (SCOPE) [24–27] and the Systolic Hypertension in the Elderly Program (SHEP) showed no significant differences between subjects provided active agents (candesartan/hydrochlorothiazide (SCOPE); chlorthalidone, atenolol, or reserpine (SHEP)) compared to placebo controls, although further analyses showed that most of the placebo controls were concurrently taking AHTs [28–31]. A more recent population study of 54 patients taking CCBs, 59 patients on angiotensin converting enzyme (ACE) inhibitors, 81 on *β*-blockers, and 16 on diuretics followed from ages 85 to 90 showed that patients on CCBs but not other AHTs had a slower decline in MMSE score (0.4 MMSE units/year) compared to control subjects not on AHTs [32]. The most recent analysis of data from the Cache County Study suggests that thiazide and potasssium sparing diuretics were associated with decreased risk of AD [33]. More recently, meta-analysis of data from 10 studies of the relationship between CCBs and later cognitive decline showed no clear evidence that CCB use increased or decreased the risk of cognitive decline or dementia in the very elderly [34].

In contrast to previous studies that evaluated the risk of AD and use of AHTs (including those in the recent meta-analyses) our current study examined the potential effects of AHTs on progression to dementia in a well-characterized group of subjects rather than assessment of risk. Our results demonstrate that use of CCBs but not other groups of AHTs significantly decreases the rate of progression of subjects to dementia compared to subjects who took no AHTs. In addition, and perhaps most importantly, use of CCBs mitigated the accelerated decline to dementia associated with the presence of the APOE-4 allele. Although several studies (reviewed in [33]) have suggested that diuretics, particularly potassium sparring diuretics, minimize the risk of developing AD in prospective studies, our study cohort had too few subjects who took only diuretics or angiotensin II receptor blockers for inclusion in the analyses.

Although AHTs appear to significantly alter the risk of development of AD, the exact mechanism remains unclear and is possibly a combination of cardiovascular effects coupled with potential effects on the pathogenic mechanisms of AD.

To examine the potential effects of CCBs on amyloid processing we treated H4 cultures with multiple CCBs and assessed cell survival and the effects of each drug on A*β*_1–42_ production. Our data show that of the CCBs tested nifedipine provided the most pronounced reduction (~40%) in A*β* production coupled with the least toxicity. To determine if nifedipine treatment significantly altered proteins associated with A*β* production we examined levels of proteins involved in A*β* processing. Our data show that nifedipine treatment significantly decreased levels of key components of the gamma secretase complex (PS-1, NCT). In addition, nifedipine significantly increased levels of ADAM-10, a presumed alpha secretase. These data are in contrast with those of Zhao et al. [35] who showed no A*β*_1–42_ lowering effects of common antihypertensive drugs although they found significant effects of furosemide (diuretic), nitrendipine (CCB), candesartan (angiotensin II receptor antagonist), and diazoxide (vasodilator) against A*β*_1–42_ oligomerization.

Although cell culture data are often difficult to extrapolate to human subjects in the clinic, our data suggest that a possible additional mechanism of action of nifedipine is through modulation of A*β* production. Clearly, further studies are needed to determine if CCB use correlates with decreased amyloid plaque deposition in AD subjects.

Modulation of A*β* has been the focus of considerable study as a potential therapeutic target in recent years. A*β* is formed by sequential cleavage of the amyloid precursor protein (APP) by the *β*-site APP cleaving enzyme (BACE), an aspartyl protease, to form the N-terminal of A*β*. The resulting C-terminal fragment of APP is cleaved at multiple sites by the *γ*-secretase complex that consists of presenilin-1 (PS-1), nicastrin (NCT), presenilin enhancer-2 (Pen-2), and APH-1 [36] to form A*β* peptides ranging from 37 (A*β*_37_) to 43 (A*β*_43_) residues. In contrast, normal processing of APP by *α*-secretase (presumed ADAM-10) leads to cleavage of APP in the middle of the A*β* sequence and the formation of secreted APP (sAPP*α*), a neurotrophic molecule. Increased processing of APP by BACE and *γ*-secretase leads to production of A*β* fragments that form neurotoxic oligomers and fibrils before final aggregation as SP [37, 38]. Multiple studies demonstrate that oligomeric A*β* inhibits long term potentiation and memory when injected into rat brain [4, 39, 40] and that oligomeric A*β* levels correlate with memory deficits in transgenic mouse models that express mutant APP, PS1, or their combination [41, 42]. Together these data have been used to support studies of potential therapeutics that modify A*β* formation including the development of BACE inhibitors and inhibitors of the *γ*-secretase complex.

Additional therapeutics aimed at minimizing A*β* processing have focused on modulation of *γ*-secretase. Inhibition of *γ*-secretase diminishes A*β* formation, prevents A*β* aggregation, and reverses APP induced cognitive deficits in transgenic models of A*β* deposition [43]. Unfortunately, *γ*-secretase functions in the cleavage of multiple transmembrane proteins in addition to APP, in particular the Notch family of transmembrane receptors required for Notch signaling [44]. Prolonged dosing of *γ*-secretase inhibitors leads to inhibition of Notch signaling and changes in the GI tract, spleen, and thymus that likely limit the extent of A*β* inhibition [45–47]. Despite this potentially serious problem, *γ*-secretase inhibitors including LY-50139 (Lilly [48, 49]) and MK0752 (Merck [50]) have been tested in humans. Unfortunately, the clinical trials have not been successful (reviewed [51]).

More recent pharmacologic approaches aimed at enhanced A*β* degradation have mainly focused on vaccinations. Initial active vaccination trials using Elan's AN1702 antibody against A*β*_42_ aggregates led to meningeal encephalopathy in 6% of the vaccinated patients and early termination of the studies [52]. More recent studies have focused on passive immunization with anti-A*β* antibodies. In transgenic models of A*β* deposition, passive immunization led to decreased plaque burden [53] and reversal of cognitive deficits [54]. Although passive immunization likely limits encephalopathies associated with active immunization, increased vascular amyloid has been observed in transgenic models [55–57]. Bapineuzumab (Elan/Wyeth), a humanized monoclonal antibody (reviewed [58]) that binds the N-terminal region of A*β* and is therefore unlikely to recognize monomeric or oligomeric A*β*, and solanezumab (Eli Lilly), a humanized A*β* antibody that binds monomeric but not fibrillar A*β*, recently failed to meet primary efficacy goals in mild to moderate AD patients although pooled analyses suggest that solanezumab may provide cognitive benefits (reviewed [59]).

Overall, the current data suggest that use of calcium channel blocker antihypertensives significantly slows the rate of progression of subjects to dementia compared to those subjects who do not use CCBs and that the potential protective effects on cognitive decline might be mediated through modulation of proteins involved in A*β* production. The current data coupled with existing risk assessment studies suggest that use of AHTs may significantly alter AD risk/progression and that use of these drugs should be considered in clinical trials of anti-AD therapeutics.

## Figures and Tables

**Table 1:** Commonly prescribed antihypertensive drugs.

Antihypertensive category	Drugs
Angiotensin converting enzyme (ACE) inhibitors	Benazepril (Lotensin), captopril (Capoten), enalapril (Vasotec), fosinopril (Monopril), lisinopril (Prinivil, Zestril), moexipril (Univasc), quinapril (Accupril), ramipril (Altace), and trandolapril (Mavik)

Angiotensin II receptor blockers	Candesartan (Atacand), irbesartan (Avapro), losartan (Cozaar), telmisartan (Micardis), and valsartan (Diovan)

Beta blockers	Acebutolol (Sectral), atenolol (Tenormin), betaxolol (Kerlone), bisoprolol (Zebeta), carteolol (Cartrol), carvedilol (Coreg), labetalol (Normodyne, Trandate), metoprolol (Lopressor, Toprol), nadolol (Corgard), penbutolol (Levatol), propranolol (Inderal), and timolol (Blocadren)

Calcium channel blockers	Amlodipine (Norvasc), clevidipine (Cleviprex), dilitiazem (Cardizem), felodipine (Plendil), isradipine (Dynacirc), nifedipine (Adalat, Procardia), nicardipine (Cardene), nimodipine (Nimotop), and nisoldipine (Sular)

Diuretics	Acetazolamide (Diamox), chlorthalidone (Thalitone), hydrochlorothiazide (HydroDiuril), indapamide (Lozol), and metolazone (Zaroxolyn, Mykrox)

**Table 2:** Subject demographic data for subjects involved in the logistic modeling study. There were insufficient numbers of subjects taking diuretics or angiotensin II receptor blockers to allow meaningful comparisons.

	CCBs			ACE			Beta blockers		
	Users	Matched nonusers	All	Users	Matched nonusers	All	Users	Matched nonusers	All
*N*	32	31	63	13	13	26	22	21	43

Initial age (mean ± SEM y)	75.4 ± 1.2	75.4 ± 1.2	75.4 ± 0.8	77.5 ± 1.6	76.9 ± 1.7	77.2 ± 1.1	75.5 ± 1.7	76.1 ± 1.7	75.8 ± 1.2

Age/pseudoage at drug initiation (mean ± SEM y)	79.2 ± 1.3	78.8 ± 1.3	79.0 ± 0.9	81.1 ± 2.1	81.1 ± 1.6	81.1 ± 1.6	79.4 ± 1.8	79.4 ± 1.8	79.2 ± 1.2

% women	73.5	75.0	74.2	61.5	61.5	61.5	52.4	54.2	52.4

Baseline comorbidities (*n* [%])									
Heart attack	3 (9.4)	1 (3.2)	4 (6.4)	1 (7.7)	0 (0.0)	1 (4.0)	3 (13.6)	0 (0.0)	3 (7.0)
Hypertension	18 (56.3)	6 (20.0)	24 (38.71)	3 (23.1)	0 (0.0)	3 (12.0)	6 (27.3)	0 (0.0)	6 (14.0)
Diabetes	3 (9.4)	1 (3.2)	4 (6.4)	2 (15.4)	1 (8.3)	3 (12.0)	1 (4.5)	2 (9.5)	3 (7.0)
High cholesterol	5 (15.6)	3 (10.3)	8 (13.11)	1 (7.7)	0 (0.0)	1 (4.0)	4 (18.2)	1 (4.8)	5 (11.6)

**Table 3:** Statistical parameters based on fit of the model to the data.

Parameter	Estimate	Standard error	DF	*t* value	Pr > |*t*|
Intercept *b*	0.1253	0.01288	62	9.72	<0.0001
Intercept *c *	17.0396	8.0292	62	2.12	0.0378
Case versus control	3.6125	1.3639	62	2.65	0.0102
APOE-4	−3.8579	1.4064	62	−2.74	0.0079
Initial age	1.0278	0.1039	62	9.89	<0.0001
Between var.	15.1200	0.9212	62	16.41	<0.0001
Within var.	15.3807	3.3457	62	4.60	<0.0001
